# Low-carbon energy transition multi-agent network evolutionary under carbon trading scheme

**DOI:** 10.1371/journal.pone.0300202

**Published:** 2024-04-25

**Authors:** Zijie Wei, Heng Wang, Tao Fang, Zhixin Han, Pengyu Wang

**Affiliations:** 1 Longyuan (Beijing) Carbon Asset Management Technology Co., Ltd, Xicheng, Beijing, China; 2 State Energy Group Henan Electric Power Co., Ltd., Zhengzhou, China; 3 School of Economics and Management, Wuhan University, Wuhan, China; 4 Research Center for Complexity Science and Management, Wuhan University, Wuhan, China; National Technical University of Athens: Ethniko Metsobio Polytechneio, GREECE

## Abstract

Transitioning to low-carbon energy is key for reaching carbon neutrality and modernizing our energy systems, but it presents significant cost-related challenges for energy businesses. To foster optimal outcomes, this paper develops a game model including power generators, high-energy businesses, and consumers in the carbon trading framework. The model explores how different entities evolve their low-carbon strategies under social learning influence to optimize utility. Stability analysis of strategy and simulation experiments reveal the following findings: (1) Greater carbon quotas reduce power generators’ low-carbon transition willingness while high-energy-consuming enterprises and consumers remain unchanged. (2) Higher prices for low-carbon products offered by high-energy-consuming enterprises boost low-carbon transition motivation across all parties. (3) Increased green premiums enhance revenue for all parties but are constrained by policy and carbon pricing. (4) Both direct and indirect increases in carbon emissions negatively impact the revenue and utility for all stakeholders. (5) Increasing social learning effect fosters a shift towards low-carbon strategies, accelerating the attainment of game equilibrium, and enhancing market stability and sustainability. This research provides decision support for carbon trading policy design and low-carbon transition of energy enterprises.

## 1. Introduction

In the 21st century, global energy security and environmental protection have become increasingly prominent issues. Advocating for a low-carbon economy, reducing fossil fuel combustion, and limiting greenhouse gas emissions have become a global consensus [1–3]. President Xi Jinping stated during the 75th session of the United Nations General Assembly on September 22, 2020, that "China will enhance its nationally determined contributions and take more forceful policies and measures. We aim to have CO2 emissions peak before 2030 and achieve carbon neutrality before 2060." In the report of the 20th National Congress of the Communist Party of China in 2022, it was emphasized that "promoting the green and low-carbon transformation of economic and social development is a key link in achieving high-quality development." The National Development and Reform Commission and the National Energy Administration also proposed in the "Opinions on Improving the Institutional Mechanism and Policy Measures for Energy Green and Low-Carbon Transformation" to "encourage industrial enterprises to carry out clean energy substitution, reduce carbon emissions per unit of product, and encourage qualified enterprises to take the lead in forming a low-carbon and zero-carbon energy consumption model." Energy green and low-carbon transformation has been widely recognized as an important strategic direction for business development [4].

Carbon markets play a crucial role in promoting energy and economic transition towards a low-carbon trajectory by utilizing market-based mechanisms to achieve the lowest emission reduction costs [5]. Through carbon market transactions, various entities can optimize the allocation of carbon emission rights through market mechanisms, thereby reducing carbon emissions [6]. However, the operation of carbon markets involves the decision-making and behaviors of multiple actors, including power generation enterprises, high-energy-consuming industries, and consumers. From the perspective of enterprises, they can promote the evolution of green and low-carbon transformation through green technology innovation and carbon emission reduction [7]. From the standpoint of consumers, they can drive the evolution of green and low-carbon transformation by purchasing low-carbon products and supporting environmentally friendly businesses [8]. These actors interact with each other in carbon market transactions, jointly promoting the transition to a low-carbon economy. Therefore, it is necessary to study the evolutionary dynamics of multi-agent low-carbon transition in carbon market transactions and clarify the underlying mechanisms and patterns behind the decision-making and behaviors of these actors.

Carbon trading policy research is one of the current hot research topics, mainly focused on the impact of carbon trading policy on the electricity market, the economy, the environment, energy, and environmental investment [9–14]. However, existing studies mainly focus on the macro-level impacts of carbon trading, often neglecting to delve into the intricacies of decision-making processes among participants within the carbon market. The evolution of multi-agent low-carbon transition has received significant attention, and scholars have conducted extensive discussions and analyses, resulting in many valuable research findings. Among them, most studies adopt the evolutionary game theory approach by constructing evolutionary game models to study the evolutionary paths and patterns of decision-making by the agents [15–20]. Evolutionary game theory, as a dynamic theory of the evolution of collective characteristics based on individual learning and selection in the interaction process, has become an essential tool for studying the agents’ decision-making evolution mechanisms. However, the successful transformation of multi-agent green and low-carbon transition requires all agents’ joint efforts and collaboration. In this process, agents must understand each other’s needs, capabilities, and limitations to better coordinate actions and resources to achieve common goals. Social learning within a social network refers to the process by which individuals or groups acquire knowledge and experience from others or the environment through observation, imitation, and communication in a network composed of various relationships and interactions. Social learning can help agents acquire this information, enabling a better understanding of each other’s roles and interests [21, 22]. However, existing research on multi-agent low-carbon transition has yet to consider the influence of social learning among agents on the decision-making process of the low-carbon transition.

Based on the above discussions, this study constructs a network evolutionary game model involving power generation enterprises, high-energy-consuming industries, and consumers in carbon market transactions to investigate the evolutionary paths and patterns of multi-agent low-carbon transition decisions under the influence of social learning. In this model, power generation enterprises, high-energy-consuming industries, and consumers adjust their low-carbon transition strategies through social learning in the social network to maximize their utilities. Furthermore, simulation analyses explore stable equilibrium strategies and key influencing factors in multi-agent low-carbon transition decision-making. The main contributions of this study are as follows: (1) revealing the game relationships between multiple agents in carbon market transactions and their impact on low-carbon transition; (2) elucidating the influence mechanisms of social learning on decision-making in multi-agent green and low-carbon transition; and (3) analyzing factors influencing the mechanisms of multi-agent low-carbon transition from multiple levels and perspectives.

## 2. Literature review

This study investigates the mechanisms of multi-agent low-carbon transition within the context of carbon market transactions. To achieve this, relevant literature is reviewed.

In previous research, scholars have examined various facets related to carbon trading and low-carbon transitions, encompassing topics such as the impact on electricity markets, economic implications, and environmental investments. For instance, Blyth et al. (2014) discussed decarbonization of electricity generation and its effects on market risks and revenue, revealing its influence on investment decisions by power generation companies. Consequently, the design of carbon markets must address issues of market power and subsidy negotiations [9]. Zhang et al. (2018) utilized dynamic recursive computable general equilibrium (CGE) models to investigate the optimal allocation of carbon quotas within the power industry. Their findings highlighted the direct impact of quota allocation on electricity prices and underscored the benefits of historical emission-based quota allocation in terms of societal and economic interests [10]. Lin and Jia (2019) employed dynamic recursive computable general equilibrium models to analyze the broader effects of carbon trading on the economy, environment, and energy. Their research demonstrated that carbon trading can significantly affect electricity prices and carbon emissions but may have adverse implications for GDP [11]. Zhang et al. (2020) employed data envelopment analysis (DEA) optimization model to assess the economic and environmental outcomes of carbon trading across various industries in China [12]. Furthermore, Zhang and Zhang (2020) constructed a carbon emissions model for the power industry, highlighting the effectiveness of carbon trading in reducing emissions compared to command-and-control policies and its additional environmental health benefits [13]. Sun et al. (2022) developed a comprehensive energy system model encompassing electricity, carbon trading, and natural gas, employing artificial intelligence algorithms to optimize the synergy among multiple systems, thereby achieving carbon emission reduction and economic benefits [14].

Additionally, research on multi-agent low-carbon transition mechanisms has been primarily grounded in evolutionary game theory. For instance, Nie et al. (2022) explored the interaction between purchasing decisions made by public transportation operators and government policy implementation decisions within the framework of carbon trading subsidy policies. They sought to identify stable strategies for all parties involved through advancements in evolutionary game theory [15]. Tian and Sun (2022) delved into the intricate dynamics among producers, regulatory agencies, third-party certification agencies, and consumers, employing a four-party evolutionary game model to elucidate their evolutionary paths and stable strategies [16]. Wang et al. (2022) introduced emotional factors as irrational elements and combined them with evolutionary game theory and RDEU theory to construct an evolutionary game model involving government regulators and energy consumers. Their analysis explored how decision-makers low-carbon emotions influence their strategy choices and energy structural transformations [17]. Yuan and Zheng (2022) applied evolutionary game theory to construct a three-party evolutionary game model involving government, enterprises, and consumers, studying the strategic choices of the these entities during low-carbon technology innovation and investigating equilibrium point stability [18]. Moreover, Li et al. (2023) examined the micro-market behavior of new energy generation companies within the context of renewable energy consumption responsibility weighting policies. Their research introduced a dual-market bidding model and a two-stage composite difference evolutionary game algorithm to address complex multi-party game scenarios [19]. Liu et al. (2023) studied the equilibrium states, evolutionary trajectories, and critical conditions of government and coal-fired power generation companies adopting carbon capture, utilization, and storage (CCUS) technology, employing evolutionary game theory and numerical analysis [20].

Furthermore, research on social learning has examined its impact on production decisions, social welfare, policy implementation, and market responses. Darby (2006) conducted a survey of British village residents and found that social learning significantly improves energy efficiency and conservation efforts [21]. Reichenbach and Requate (2012) investigated the influence of learning-by-doing, spillover effects, and other factors on the production decisions of renewable energy generators, quantifying the welfare loss associated with feed-in tariffs [22]. Squires and Vestergaard (2018) argued that market policy tools alone might not effectively address public issues or achieve optimal economic welfare. They used real-life cases to illustrate the impact of technological transformation, knowledge spillovers, and social learning [23]. Shi et al. (2021) constructed an evolutionary game model based on social networks to investigate the influence of carbon taxes, subsidy negotiations, and low-carbon product demand within the context of social learning [24]. Kang et al. (2021) developed a data-driven social learning model, which highlighted the role of moderate publicity in completing projects related to carbon emission reduction policies [25]. Wang et al. (2022) examined the role of social learning processes among consumers in the electricity retail market, particularly in peak load shifting through time-of-use pricing. Their findings emphasized the positive impact of social learning behaviors on market competition and the ability of retailers to balance electricity demand [1]. Cheng et al. (2021) conducted a study on the influence of social learning on the adoption of low-carbon lifestyles among urban residents in China, affirming the increasing importance of social learning in policy implementation [26]. Gong and Diao (2023) considered two modes of social learning and pointed out that investor interactions based on networks contribute to market stability [27].

However, existing research has predominantly concentrated on the macro-level impacts of carbon trading, often neglecting to delve into the intricacies of decision-making processes among market participants in response to various policies or evaluate the influence of social learning among market participants on the decision-making process related to the low-carbon transition. Therefore, it is essential to develop a network evolutionary game model that incorporates power generation companies, high-energy-consuming enterprises, and consumers engaged in carbon market transactions. This model aims to explore the evolutionary paths and patterns governing multi-agent low-carbon transition decision-making under the influence of social learning. Additionally, it seeks to conduct simulation analysis to examine stable equilibrium strategies and key influencing factors governing multi-agent low-carbon transition decisions. This study bridges the existing research gap by revealing the mechanisms of influence among stakeholders through the utilization of real-world data. The model’s effectiveness will be validated, providing decision support for enterprise embarking on low-carbon transition initiatives.

### 3. Model assumptions and evolutionary rules

#### 3.1. Model assumptions

With the increasing severity of global climate change, governments have recognized the urgency of low-carbon energy transition and formulated policies to achieve carbon peak and carbon neutrality targets. However, power generation companies, high-energy-consuming enterprises, and ordinary consumers face significant challenges in this process. The high costs of low-carbon transition and the presence of externalities make it difficult for these enterprises to progress in the transition [28]. At the same time, consumers are concerned about the stability and cost of the new energy supply. To find a suitable path for low-carbon transition in this complex social environment, this paper constructs a network evolutionary game model under the carbon trading framework. This model includes power generation companies, high-energy-consuming enterprises, and consumers as three key stakeholders. It explores how multiple agents make decisions and maximize their utilities through social learning during the low-carbon transition process. The relevant assumptions of the model are as follows:

Assumption 1: The set of power generation companies is denoted as *i* ∈ *Gen* = {1,2,⋯,*N*_*g*_}, the set of high-energy-consuming enterprises is denoted as *j* ∈ *Fim* = {1,2,⋯, *N*_*c*_}, and the set of consumers is denoted as *k* ∈ *Com* = {1,2,⋯, *N*_*u*_}. There exists a social network among power generation companies, high-energy-consuming enterprises, and consumers.


$$A_{g}={\left[a_{ij}^{g}\right]}_{N_{g}\times N_{g}},A_{c}={\left[a_{ij}^{c}\right]}_{N_{c}\times N_{c}},A_{u}={\left[a_{ij}^{u}\right]}_{N_{u}\times N_{u}}$$ (1)

There exists a trading network between power generation companies and high-energy-consuming enterprises:

$$B_{gc}={\left[b_{ij}^{gc}\right]}_{N_{g}\times N_{c}}$$ (2)

There exists a trading network between high-energy-consuming enterprises and consumers:

$$B_{cu}={\left[b_{ij}^{cu}\right]}_{N_{c}\times N_{u}}$$ (3)

Where $a_{ij}^{g},a_{ij}^{c},a_{ij}^{u},b_{ij}^{gc},b_{ij}^{cu}\in \left\{0,1\right\}$, with 0 indicating no relationship and 1 indicating the presence of a relationship.

Assumption 2: Due to the significant costs and technological barriers associated with low-carbon transformation strategies [29, 30], both high-energy-consuming enterprises and power companies face substantial uncertainty when choosing such strategies [16, 31, 32]. This uncertainty leads to a lack of incentive for them to compromise their current self-interest in reducing carbon emissions [33]. Furthermore, even if high-energy-consuming enterprises and power companies initiate low-carbon transformation plans, consumers may still be reluctant to opt for low-carbon products due to their higher prices [34]. Therefore, in this paper, we assume that consumers *k* ∈ {1,2,⋯, *N*_*u*_} have two purchasing strategies: buying low-carbon products (BL) represented by *su*_*k*_ = 1, and buying traditional products (BH) represented by *su*_*k*_ = 0. High-energy-consuming companies *j* ∈ {1,2,⋯, *N*_*c*_} have two strategies: low-carbon transformation (CLT) represented by *sc*_*j*_ = 1, and non-low-carbon transformation (NCLT) represented by *sc*_*j*_ = 0. Power generation companies *i* ∈ {1,2,⋯, *N*_*g*_} have two strategies: low-carbon transformation (GLT) represented by *sg*_*i*_ = 1, and non-low-carbon transformation (NGLT) represented by *sg*_*i*_ = 0.

Assumption 3: The quantity of products consumers purchase depends on their strategy and the strategy of high-energy-consuming companies. If a high-energy-consuming company offers low-carbon products and a consumer chooses the BL strategy, they will purchase a quantity *Q*_1_ of products at a price of *P*_1_. If a high-energy-consuming company does not offer low-carbon products and a consumer chooses the BH strategy, they will purchase a quantity *Q*_2_ of products at a price of *P*_2_. When other high-energy-consuming companies cannot provide satisfactory products to consumers, the quantity consumer purchases is zero. Therefore, the amount of goods purchased by consumer *k* from high-energy-consuming company *j* is represented as:

$$Q_{kj}=\left(s_{k}s_{j}Q_{1}+\left(1-s_{k}\right)\left(1-s_{j}\right)Q_{2}\right)b_{jk}^{cu}$$ (4)

The low-carbon transformation of high-energy-consuming company *j* can bring environmental benefits *R* to consumers. Additionally, the intrinsic value that consumers derive from each unit of product is represented as *U*. Furthermore, consumers can gain an additional psychological value *V* from purchasing low-carbon products.

Assumption 4: High-energy-consuming company j incurs a certain transformation cost *C*_2_ when choosing the CLT strategy. Additionally, due to advanced technological support, the company has sufficient production flexibility to respond to market demands at any time quickly. Therefore, without loss of generality, we assume that the production cost for high-energy-consuming companies is standardized, i.e., cost equals zero. Based on Assumption 3, the product demand faced by high-energy-consuming company *j* is represented as:

$$Q_{j}=\sum_{k=1}^{N_{u}}Q_{kj}$$ (5)

And the electricity consumption per unit of production is represented as *q*. Let $D_{ij}=qQ_{j}b_{ij}^{gc}$ represent the electricity quantity that high-energy-consuming company *j* purchases from electricity generation company *i*, where the electricity price is dependent on the strategy chosen by the generation company. When generation company *i* chooses the GLT strategy, the high-energy-consuming company must pay a certain green premium. Let *G*_1_ represent the electricity price when generation company *i* chooses the GLT strategy, and let *G*_2_ represent the electricity price when generation company *i* chooses the NGLT strategy. When high-energy-consuming company *j* chooses to produce low-carbon products, the carbon emissions per unit of production are represented as *t*_1_. Conversely, when high-energy-consuming company *j* chooses not to produce low-carbon products, the carbon emissions per unit of production are represented as *t*_2_. Therefore, the carbon emissions of high-energy-consuming company j are represented as:

$$E_{j}=\left(t_{1}s_{j}+\left(1-s_{j}\right)t_{2}\right)Q_{j}$$ (6)

Assumption 5: Generation company *i* incurs a certain transformation cost *C*_3_ when choosing the GLT strategy. Similarly, we can standardize the production cost of the generation company to zero. Based on assumption 4, the electricity demand faced by generation company *i* is represented as $D_{i}=\sum_{j=1}^{N_{c}}Q_{ij}$. When generation company *i* chooses the GLT strategy, the carbon emissions per unit of electricity generation are represented as *h*_1_. Conversely, when generation company *i* chooses not to adopt the GLT strategy, the carbon emissions per unit of electricity generation are represented as *h*_2_. Therefore, the carbon emissions of generation company *i* are represented as:

$$E_{i}=\left(h_{1}s_{i}+\left(1-s_{i}\right)h_{2}\right)D_{i}$$ (7)

Assumption 6: The generation and high-energy-consuming companies need to participate in carbon market trading. Let *K*_*g*_ represent the government-allocated exempted carbon allowances for the generation company, and *K*_*c*_ represent the government-allocated exempted carbon allowances for the high-energy-consuming company. The carbon market price is determined by market supply and demand and is represented as:

$$p_{c}=a_{c}-b_{c}\left(\sum_{i}\left(K_{c}-E_{i}\right)+\sum_{j}\left(K_{g}-E_{j}\right)\right)$$ (8)

### 3.2. Evolutionary rules

In real economic activities, market entities such as high-energy-consuming enterprises, power generators, and consumers are influenced by internal and external environments, which results in information asymmetry and uncertainty in value co-creation effects when these entities make decisions on complex issues, thereby exhibiting limited rational characteristics [29, 35]. Internal environments refer to the influencing factors within the market entities, including but not limited to enterprise carbon emissions, transformation costs, and consumer low-carbon preferences. These internal environmental factors affect market entities’ decision-making on complex issues and may lead to information asymmetry and uncertainty in value co-creation effects. External environments refer to various influencing factors outside the market entities, such as carbon-electricity coordination policies, social network structures among market entities, and market competition conditions. These external environmental factors may further exacerbate the uncertainty and complexity faced by market entities when making decisions, thereby affecting their limited rational performance. In this paper, the decision environment faced by market participants is defined as including external environmental influencing factors such as changes in decisions of other market participants, product prices, green premiums, as well as internal environmental influencing factors like product demand, psychological value, production costs, and more.

Therefore, when facing complex decision environments, market participants typically tend to gather information and assist their decision-making through the use of sociological learning methods [36–38]. In this paper, sociological learning is defined as the process through which knowledge, skills, or experience are exchanged and constructed through interactions between two or more individuals [39–42]. In other words, market participants, in making decisions, need to undergo a process of observing others and selecting strategies; otherwise, it may have negative implications for management policies or economic decisions and even lead to decision failures [16]. Early research focused on learning outcomes in homogeneous societies (i.e., entities with similar preferences) and simple social structures (i.e., sequential actions and observations) [43, 44]. However, recent research has gradually broadened its focus to include heterogeneous societies and more complex social networks [45–47].

Furthermore, with the rapid development of the internet and the Internet of Things (IoT), various industries have indeed exhibited complex network characteristics [48]. These network characteristics enable businesses to influence the dissemination of their strategies and low-carbon strategies by directly exchanging information and learning through connections. Different relationships between businesses may affect their cognition and behavior, thus impacting their strategic choices [49, 50]. Similarly, for consumers, by observing and imitating the consumption strategies of other consumers, they can better adapt to the constantly changing social environment [1]. In other words, sociological learning serves as an effective tool for transferring information from existing individuals to other individuals [51, 52].

This article defines the process by which individuals or groups obtain knowledge and experience from observing, imitating, and exchanging information with others in social networks composed of various relationships and interactions as follows:

At time t, the profit of generation company *i* ∈ *Gen* is denoted as *F*_*it*_. Within the social network, there exist social relationships with generation company *i*, and the set of generation companies that have social relationships and choose the GLT strategy is denoted as $G_{i}^{+}=\left\{l|l\in Gen\;and\;a_{il}^{g}=1\;and\;{sg}_{l}=1\right\}$. Similarly, the set of generation companies that have social relationships and choose the NGLT strategy is denoted as $G_{i}^{+}=\left\{l|l\in Gen\;and\;a_{il}^{g}=1\;and\;{sg}_{l}=0\right\}$.

Therefore, at time *t* + 1, generation company *i* will choose the GLT strategy with a probability of *ρ*_*it*_ and the NGLT strategy with a probability of 1 − *ρ*_*it*_, where:

$$\rho_{it}=\frac{1}{1+exp\left(\beta_{g}\left(w_{2t}-w_{1t}\right)\right)}$$ (9)

$$w_{2t}=\frac{1}{\left|G_{i}^{-}\right|}\sum_{l\in G_{i}^{-}}F_{lt},w_{1t}=\frac{1}{\left|G_{i}^{+}\right|}\sum_{l\in G_{i}^{+}}F_{lt}$$ (10)

Similarly, at time *t* + 1, high-energy-consuming companies and consumers will also learn and observe other entities in the social network using the same principles. In this paper, market agents such as high-energy-consuming enterprises, power generation companies, and consumers adopt binary strategies, which means their strategy optimization process can be described as follows: Each agent adjusts the probability of imitation by comparing the difference in average utility between the two strategies. In other words, if the low-carbon strategy leads to a higher average utility, then in subsequent simulation processes, the probability of that agent adopting the low-carbon strategy increases. As the evolutionary process unfolds, each agent carefully observes and learns from the behaviors and utilities of others, continually updating their own strategy choices based on this information. Ultimately, the market reaches a relatively stable equilibrium. This equilibrium implies that all market agents have chosen strategies that best adapt to the current market environment, resulting in overall market stability.

## 4. Model simulation analysis

### 4.1. Initial parameter settings for simulation analysis

In this study, the aluminum electrolysis industry is taken as a representative of high-energy-consuming industries to analyze low-carbon transformation dynamics. The aluminum electrolysis industry is an important non-ferrous metal industry primarily used to produce aluminum and its alloys. Aluminum is a lightweight, corrosion-resistant, highly conductive, and recyclable metal material with wide applications in construction, transportation, aerospace, electronics, and daily life. However, the aluminum electrolysis process produces significant carbon emissions, causing adverse environmental impacts. In this section, we analyze the low-carbon transformation dynamics of power generation and aluminum electrolysis companies in the electricity-carbon market based on trading data from a specific aluminum electrolysis company and fundamental data of China’s aluminum electrolysis industry.

As a large electricity consumer, the aluminum electrolysis industry consumes 13,500 kWh per ton of aluminum produced, ranking first among major industrial products. Hence, we set *q* = 13,500. According to the operational data of the company, the price of one ton of aluminum electrolysis ranges from 12,000 to 23,000 RMB, with an average price of *P*_2_ = 14,304 RMB. The average monthly production is *Q*_2_ = 27,033 tons. Additionally, based on research conducted by the China International Capital Corporation (CICC), the green premium rate for achieving zero-emission aluminum electrolysis through direct and indirect emission reduction measures is approximately 34%, accompanied by a demand contraction of around 3%. Therefore, the transformed low-carbon aluminum electrolysis companies will sell their products at a price of *P*_2_ = 19167.36 = 14304 × 1.34 RMB, and the average monthly production will decrease to *Q*_1_ = 26222.01 = 27033 × 0.97 tons. Consumers are willing to pay around a 10% premium for low-carbon products, resulting in an environmental value of *R* = 1916.736 = 14304 × 1.34 × 0.1, and let *U* = 12000 RMB.

The carbon emissions of power generation companies can be measured using carbon emission factors, which represent the amount of carbon emissions generated per unit of electricity produced. According to the "Average Carbon Emission Factors of Chinese Regional Power Grids in 2011 and 2012," it is known that in regions of China where coal-fired power generation is dominant, the carbon emission factor is 0.8843 (*h*_2_). In areas where renewable energy is actively developed in China, the carbon emission factor is 0.6671 (*h*_1_). Furthermore, according to data from the International Aluminium Institute (IAI), the carbon dioxide emissions of aluminum electrolysis from production to final transportation are approximately 16.5 tons per ton of aluminum produced (*t*_2_). Suppose the aluminum electrolysis companies also choose to undergo a low-carbon transformation. In that case, it can be assumed that the reduction in their carbon emissions will be proportional to that of power generation companies, i.e., *t*_1_ = 11.5 = 16.5 × 0.7. The electricity price for large industrial and commercial users in China is approximately 0.5 RMB/kWh, and the green premium for renewable energy generation is about 0.1 RMB/kWh. Therefore, we can set *G*_2_ = 0.5 and *G*_1_ = 0.6.

Finally, according to the current allocation of carbon quotas in the Chinese carbon trading market for power generation companies, it is known that the allocation ratio of carbon quotas for power generation companies is approximately 0.8444. Therefore, we can set *K*_*c*_ = 4222 and *K*_*g*_ = 6755. Furthermore, as the national carbon market in China expands, the price of carbon emission allowances generally ranges from 40 to 70 RMB. Therefore, we can set *a*_*c*_ = 55 and *b*_*c*_ = 0.0001071.

**Fig 1:**
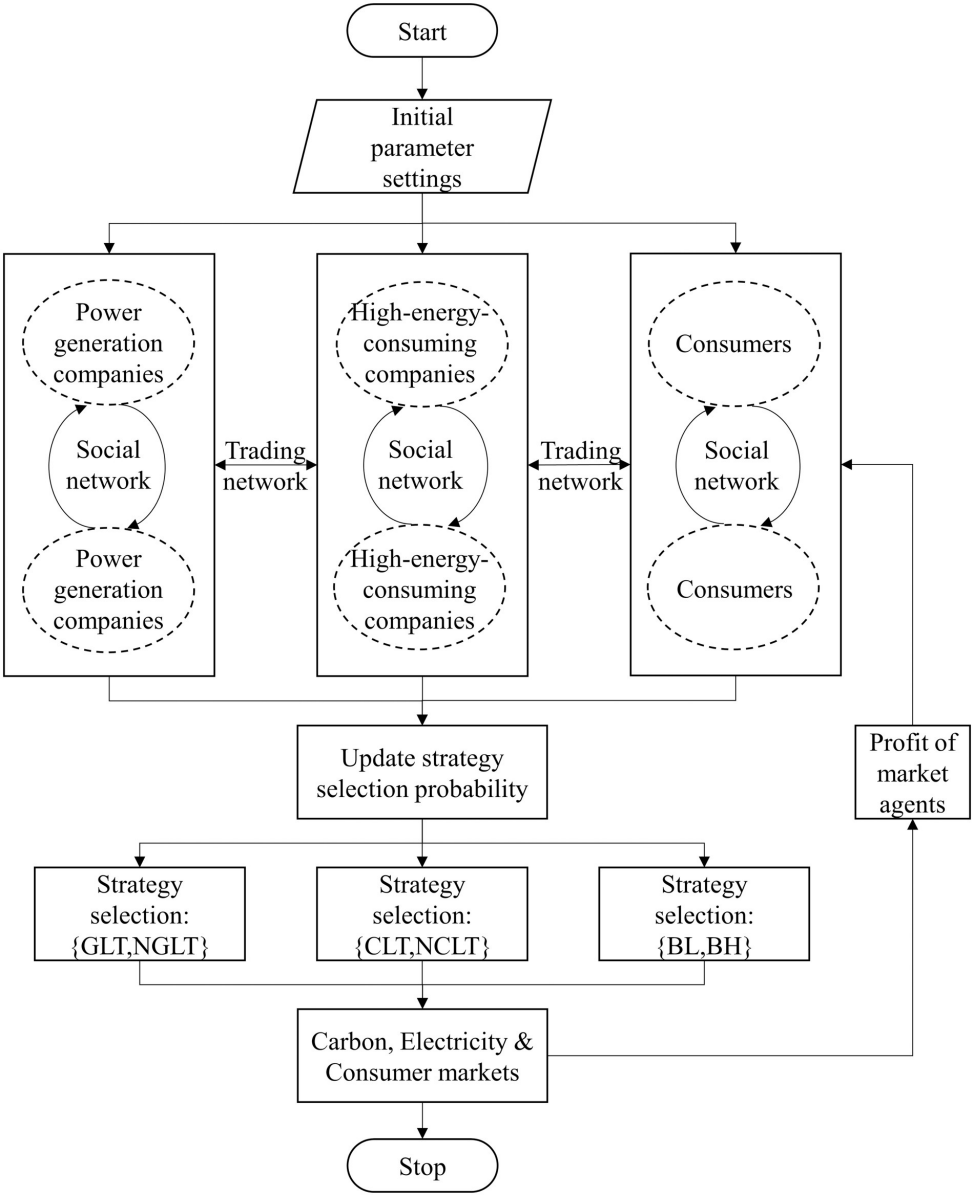
The flowchart of simulation process.

The flowchart of the simulation process is depicted in Fig 1.

### 4.2. Simulation analysis

**Fig 2:**
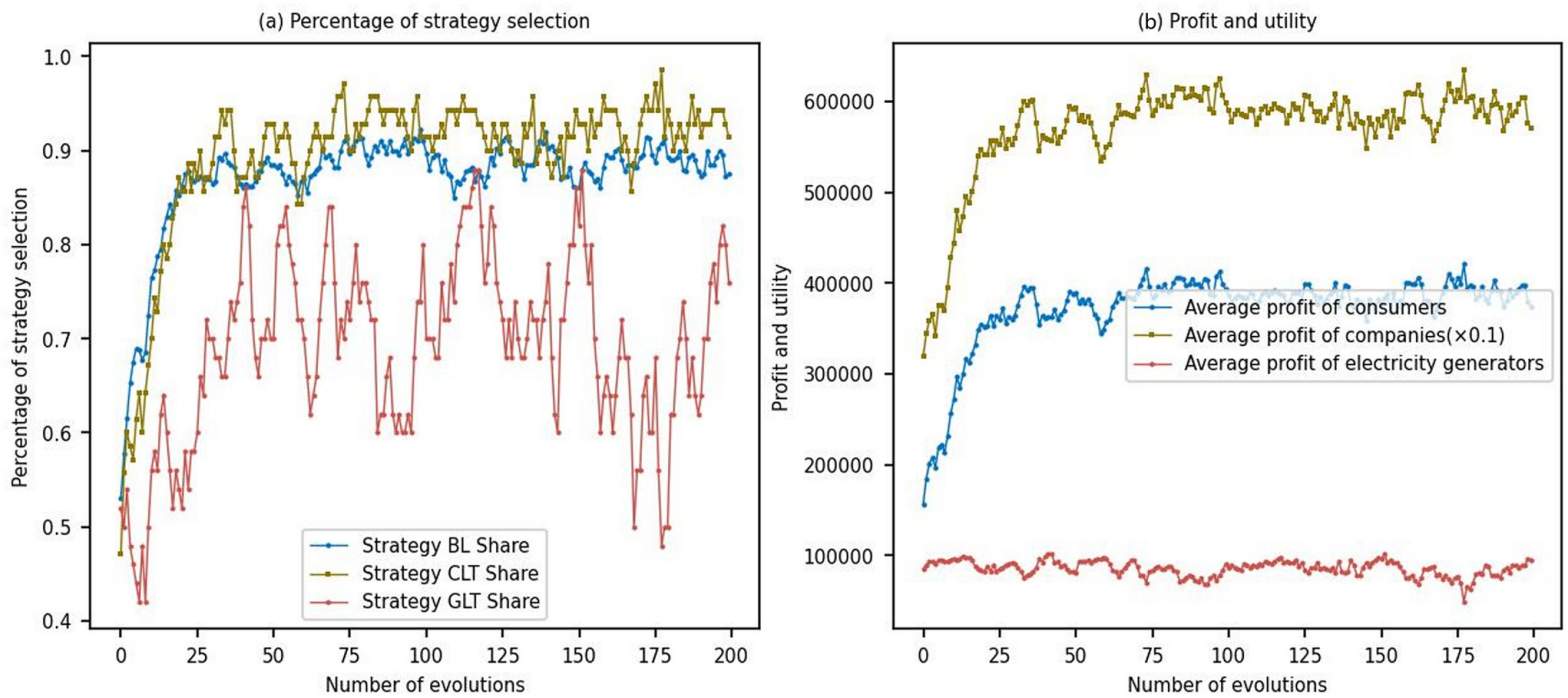
Simulation results with baseline parameters.

Based on the model parameter settings outlined in Section 4.1, the model was subjected to multiple simulations in this study. The results are depicted in Fig 2.

From Fig 2a, it can be observed that as the evolution progresses, the proportion of consumers purchasing low-carbon products gradually increases and stabilizes around 0.9. The proportion of firms opting for the CLT strategy gradually increases and approaches approximately 1. In contrast, the proportion of power generation firms selecting the GLT strategy gradually increases and fluctuates between 0.6 and 0.8.

Fig 2b shows that as the evolution unfolds, the average profits of firms and the average utility of consumers gradually increase and reach a stable state. However, the average profits of power generation firms do not significantly increase. This can be attributed to two main reasons: firstly, the limited number of power generation firms results in a smaller pool of entities available for learning, making it challenging to achieve stable strategy selection. Secondly, the relatively low green premium for electricity sales after power generation firms undergo low-carbon transformation leads to their profits being more influenced by the carbon market dynamics.

To further discuss the factors influencing multi-agent low-carbon transformation, this study analyzes aspects such as carbon quota allocation, product green premium, direct carbon emissions from products, and indirect energy-related carbon emissions.

#### Sensitivity analysis of carbon quota allocation for high-energy-consumption firms

This study conducted simulations to analyze the changes in the proportion of multi-agent strategy selections and their profits for the electrolytic aluminum company, considering varying carbon quota allocations ranging from 3000 to 7000.

**Fig 3:**
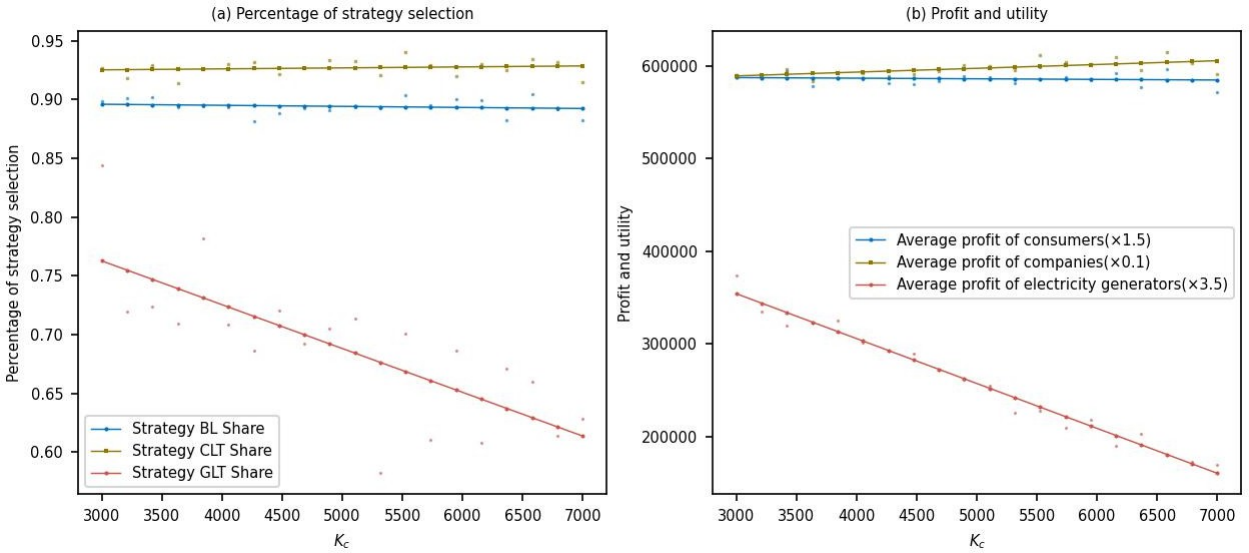
Sensitivity analysis of carbon quota allocation for high-energy-consumption firms.

Fig 3a shows that as the government increases the carbon quota allocation for the electrolytic aluminum company, the proportion of power generation companies choosing the low-carbon transformation strategy gradually decreases from approximately 0.75 to around 0.6. This indicates that when the government allocates more carbon quotas to the electrolytic aluminum company, the demand for low-carbon transformation decreases among power generation companies. This may be because they perceive the carbon emission restrictions to be relatively lenient and therefore need incentives to implement low-carbon transformation. Meanwhile, the proportion of electrolytic aluminum companies choosing the low-carbon transformation strategy remains around 0.925, indicating that these companies continue to adhere to low-carbon transformation even with the increased carbon quota allocation. This may be attributed to their long-term profit expectations from low-carbon transformation or consumers’ preference for low-carbon products. The proportion of consumers purchasing low-carbon products remains around 0.9, indicating a sustained high demand for low-carbon products.

Fig 3b shows that as the government increases the carbon quota allocation for the electrolytic aluminum company, the average profits of power generation companies decrease significantly. This is because power generation companies do not implement the low-carbon transformation strategy, leading to the need for them to purchase more carbon quotas in the carbon market, thereby increasing their costs. Meanwhile, the profits of electrolytic aluminum companies gradually increase, possibly due to their ability to sell surplus carbon quotas in the carbon market, thereby increasing their revenue. The average utility of consumers is the same, indicating that the increased carbon quota allocation for the electrolytic aluminum company does not significantly impact consumers.

In summary, under the scenario of increased carbon quota allocation by the government for the electrolytic aluminum company, the willingness of power generation companies to undergo low-carbon transformation decreases. In contrast, the behavior of electrolytic aluminum companies and consumers remains relatively unchanged. The adverse impact of increasing carbon quota allocation on the low-carbon transformation of entities is evident in a number of studies, and listed here only a few typical studies [6, 10, 16, 53]. This suggests that the government needs to adjust its carbon quota allocation strategy to ensure sufficient incentives for power generation companies to engage in low-carbon transformation. Additionally, the government should consider strengthening regulatory oversight of electrolytic aluminum companies to ensure they actively undertake low-carbon transformation measures while enjoying increased carbon quotas.

#### Sensitivity analysis of carbon quota allocation for power generation companies

This study conducted a sensitivity analysis on the carbon quota allocation for power generation companies, with carbon quotas ranging from 6000 to 10000. The simulation examined the variations in the proportions of strategy choices and their corresponding profits among multiple stakeholders.

**Fig 4:**
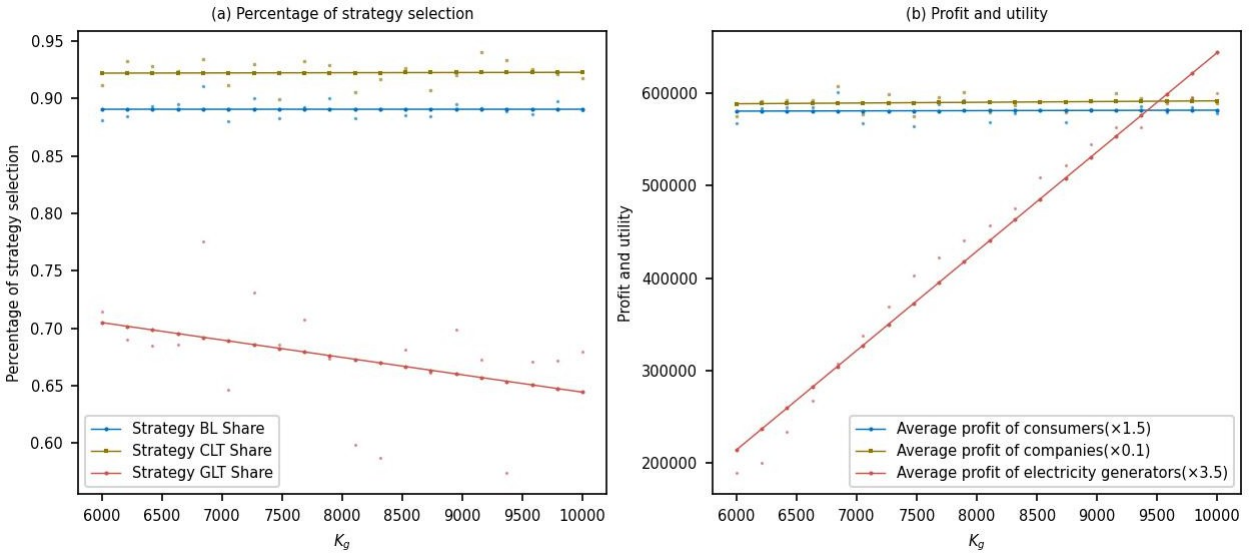
Sensitivity analysis of carbon quota allocation for power generation companies.

Fig 4a shows that as the government increases the carbon quota allocation for power generation companies, the proportion of power generation companies choosing the low-carbon transformation strategy gradually decreases from around 0.7 to 0.65. This indicates a decrease in their enthusiasm for low-carbon transformation when given more carbon quotas by the government. This could be attributed to the perception of lower urgency for low-carbon transformation when carbon emission restrictions are relatively lenient, reducing willingness to invest in low-carbon transformation. This conclusion aligns with findings observed when additional carbon quotas are allocated to aluminum electrolysis companies by the government. Meanwhile, the proportion of aluminum electrolysis companies choosing the low-carbon transformation strategy slightly increases and remains around 0.925. This suggests that aluminum electrolysis companies continue to adhere to low-carbon transformation, possibly due to their recognition of long-term benefits or consumer preferences for low-carbon products. The proportion of consumers purchasing low-carbon products remains stable at around 0.88, indicating a consistent demand for low-carbon products.

Fig 4b shows that as the government increases the carbon quota allocation for power generation companies, the average profits of power generation companies significantly increase. This is likely due to the relatively lenient carbon emission restrictions, resulting in lower carbon emission costs for power generation companies and thus improving their profits. Meanwhile, there are no significant changes in the profits of aluminum electrolysis companies and the average utility of consumers, which could be attributed to their continued adherence to low-carbon transformation and purchasing behavior under the influence of carbon quota allocation.

From an economic perspective, the government needs to balance the demands of different stakeholders when allocating carbon quotas. While providing more carbon quotas to power generation companies, the government should ensure their enthusiasm for low-carbon transformation is not compromised [10]. Additionally, the government should also pay attention to the behavior of aluminum electrolysis companies and consumers, ensuring their continued commitment to low-carbon transformation and purchasing of low-carbon products while adjusting carbon emission policies.

#### Sensitivity analysis of low-carbon product price

This study conducted simulations to analyze the changes in the proportion of strategy choices and the corresponding profits among multiple stakeholders as the price of low-carbon products varied from 12000 to 20000.

**Fig 5:**
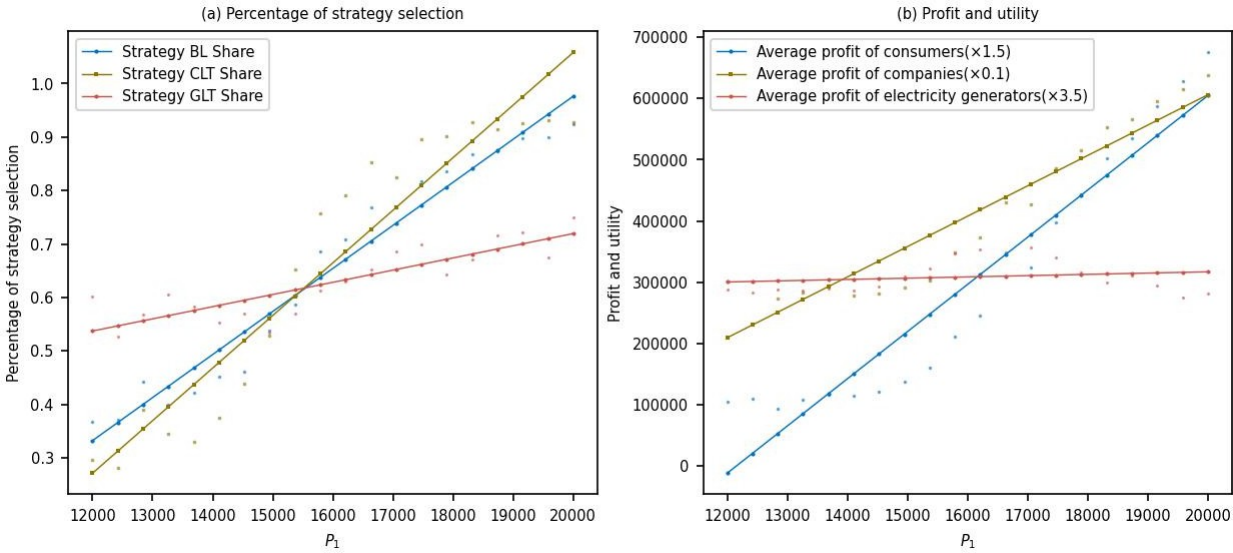
Sensitivity analysis of low-carbon product price.

Fig 5a shows that as the price of low-carbon products for aluminum electrolysis companies increases, the proportion of power generation companies choosing the low-carbon transformation strategy gradually increases from around 0.5 to around 0.7. This indicates that higher prices of low-carbon products enhance the motivation of power generation companies to engage in low-carbon transformation, possibly due to their anticipation of increased demand in the low-carbon energy market and potential profit gains. Simultaneously, the proportion of aluminum electrolysis companies choosing the low-carbon transformation strategy rapidly increases from around 0.1 to around 0.98, indicating their increased willingness to invest in low-carbon transformation to meet market demand and enhance competitiveness in response to higher prices of low-carbon products. Additionally, the proportion of consumers choosing to purchase low-carbon products gradually increases from around 0.35 to around 0.95, indicating a growing preference for low-carbon products among consumers, possibly influenced by heightened environmental awareness and improved quality of low-carbon products. Hence, a viable approach to reducing carbon emissions involves enhancing the promotion of consumers’ environmental awareness and directing them towards the purchase of low-carbon products [53].

Fig 5b shows that as the price of low-carbon products for aluminum electrolysis companies increases, there is no significant change in the average profit of power generation companies, which act as upstream players in the supply chain. This is likely because the profit of power generation companies is influenced by multiple factors, such as policies and market competition, rather than solely dependent on the price of low-carbon products. However, aluminum electrolysis companies’ profits and consumers’ average utility experience significant increases. This suggests that, against the backdrop of rising prices of low-carbon products, aluminum electrolysis companies benefit from higher profits through low-carbon transformation. At the same time, consumers enjoy better products and services, thereby increasing their utility.

In conclusion, from an economic perspective, as the price of low-carbon products for aluminum electrolysis companies increases, there is a general motivation for all stakeholders to engage in low-carbon transformation. Governments and industries should seize this trend to promote carbon reduction and the development of a low-carbon economy while also focusing on consumer demands and providing high-quality low-carbon products [53].

#### Sensitivity analysis of green premium on electricity

This study conducted simulations to analyze the variations in the proportions of multi-agent strategy selection and their corresponding profits in response to changes in the green premium on electricity, ranging from 0.5 to 1.

**Fig 6:**
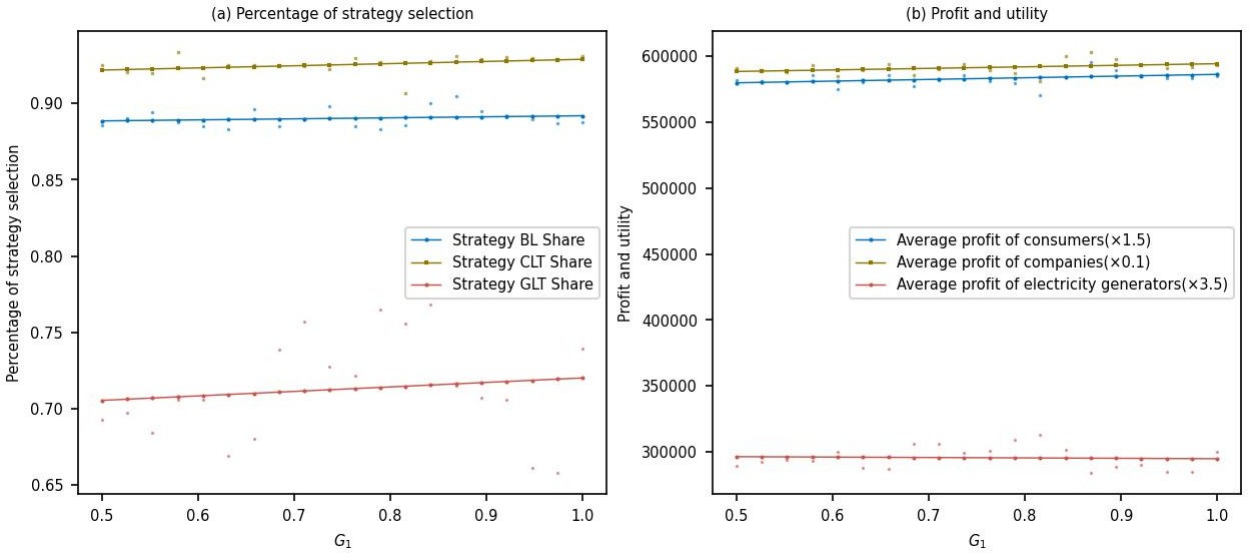
Sensitivity analysis of green premium on electricity.

From Fig 6a, it can be observed that as the green premium on electricity increases after the low-carbon transformation of power generation companies, the proportion of power generation companies choosing the low-carbon transformation strategy gradually increases from around 0.7 to approximately 0.4. This indicates that power generation companies are willing to undergo low-carbon transformation to attain higher profits in response to the rising green premium. Moreover, there is a slight increase in the proportion of aluminum electrolysis companies opting for the low-carbon transformation strategy and consumers choosing to purchase low-carbon products. This suggests increased demand for low-carbon products among aluminum electrolysis companies and consumers, potentially influenced by environmental awareness and policy advocacy.

From Fig 6b, it can be seen that with the increase in the green premium on electricity after the low-carbon transformation of power generation companies, there are no significant changes in the average profits of power generation companies and aluminum electrolysis companies, as well as the average utility of consumers. This is mainly attributed to the limited changes in the green premium on electricity after the low-carbon transformation, possibly due to policy restrictions, with the profits of power generation companies primarily influenced by carbon market prices.

From an economic perspective, the increase in the green premium on low-carbon products positively impacts the profits and utility of power generation companies, aluminum electrolysis companies, and consumers to a certain extent. However, the magnitude of these changes is limited due to policy restrictions and the impact of the carbon market prices. Therefore, the government and industry need to continue promoting the development of a low-carbon economy, optimize policies and market mechanisms, further incentivize businesses to undergo low-carbon transformations, meet consumer demand for low-carbon products, and achieve carbon reduction goals and sustainable development.

#### Analysis of the sensitivity of direct carbon emissions per unit of product

This study simulated the changes in the proportion of multi-agent strategy choices and their benefits within the range of direct carbon emissions per product unit from 5 to 16.

**Fig 7:**
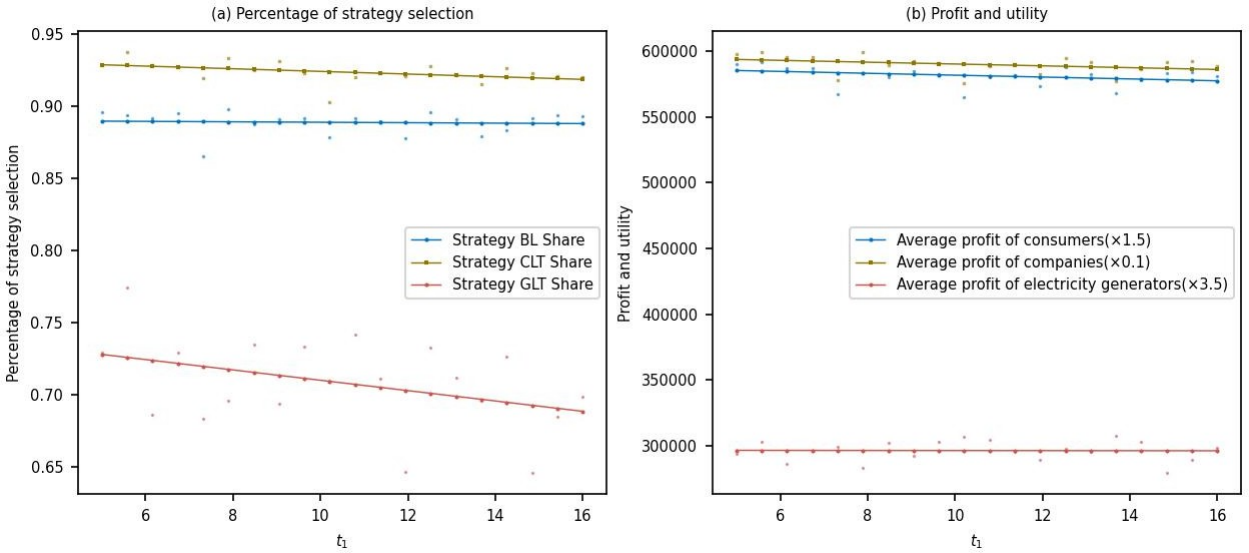
Sensitivity analysis of direct carbon emissions per unit of product.

From Fig 7a, it can be observed that as the carbon emissions per metric ton of electrolytic aluminum increase after the low-carbon transformation of aluminum smelting companies, the proportion of power generation companies choosing low-carbon transformation strategies gradually decreases from around 0.74 to approximately 0.68. This indicates that power generation companies opting for low-carbon transformation may face more significant competitive pressure in the electrolytic aluminum industry as carbon emissions rise. Additionally, there is a slight decrease in the proportion of electrolytic aluminum companies choosing low-carbon transformation strategies, which may be attributed to the higher cost of low-carbon transformation, leading some companies to temporarily need more motivation for transformation. Furthermore, there is no significant change in the proportion of consumers choosing to purchase low-carbon products, which may be related to consumers’ level of awareness and willingness to buy low-carbon products.

Fig 7b shows that as the carbon emissions per metric ton of electrolytic aluminum increase after the low-carbon transformation of aluminum smelting companies, there is no noticeable change in the average profits of power generation companies. This could be because there is still a high demand for low-carbon electricity in the market, thereby not significantly affecting the profits of power generation companies. However, the average profits of electrolytic aluminum companies and the average utility of consumers both show a declining trend. This suggests that the competitiveness of electrolytic aluminum companies may be affected by an increase in carbon emissions, and consumer welfare may be compromised due to the rise in carbon emissions.

From an economic perspective, the increase in carbon emissions per metric ton of electrolytic aluminum after the low-carbon transformation negatively impacts the profits and utility of power generation companies, electrolytic aluminum companies, and consumers. To achieve carbon reduction goals and promote green development, governments and industries should take measures to encourage low-carbon transformation in electrolytic aluminum companies. These measures may include providing policy support, investing in technological research and development, and creating market incentives. Simultaneously, raising consumer awareness and willingness to purchase low-carbon products is also essential.

#### Sensitivity analysis of carbon emissions per unit of electricity

This study simulated the changes in the proportion of multi-agent strategy choices and their benefits within the range of carbon emissions per unit of electricity from 0.2 to 0.8.

**Fig 8:**
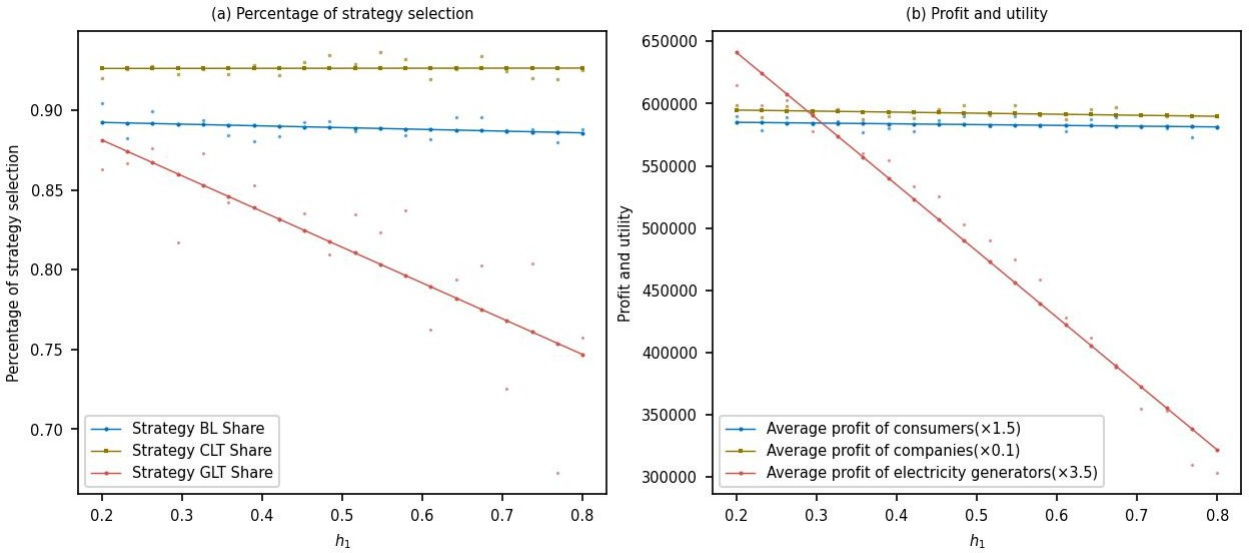
Carbon emissions per unit of electricity.

From Fig 8a, it can be observed that as the carbon emissions per unit of electricity increase after the low-carbon transformation of power generation companies, the proportion of power generation companies choosing low-carbon transformation strategies rapidly decreases from around 0.9 to approximately 0.75. This indicates that power generation companies implementing low-carbon transformation may face more significant competitive pressure as carbon emissions rise. At the same time, there is no significant change in the proportion of electrolytic aluminum companies choosing low-carbon transformation strategies. This may be because these companies face more minor challenges in low-carbon transformation than power generation companies or have already achieved some progress in low-carbon transformation. Additionally, there is a slight decrease in the proportion of consumers choosing to purchase low-carbon products, which may be related to consumers’ level of awareness and willingness to buy low-carbon products, or it could be due to the higher prices of low-carbon products, causing some consumers to hesitate in their purchases.

Fig 8b shows that as the carbon emissions per unit of electricity increase after the low-carbon transformation of power generation companies, there is a significant decline in the average profits of power generation companies. This may be because the competitiveness of power generation companies decreases in the market with increased carbon emissions, resulting in reduced demand for low-carbon electricity and, consequently, a decline in profits. The average profits of electrolytic aluminum companies and the average utility of consumers both show a slight decrease, which may be due to the negative impact of increased carbon emissions on the entire industry chain, thereby reducing the economic benefits of companies and the welfare of consumers. In conclusion, owing to the adverse environmental externalities arising from carbon emissions, both direct and indirect increments in carbon emissions have a detrimental impact on the income and utility of all stakeholders [54, 55].

#### Social learning effects

**Fig 9:**
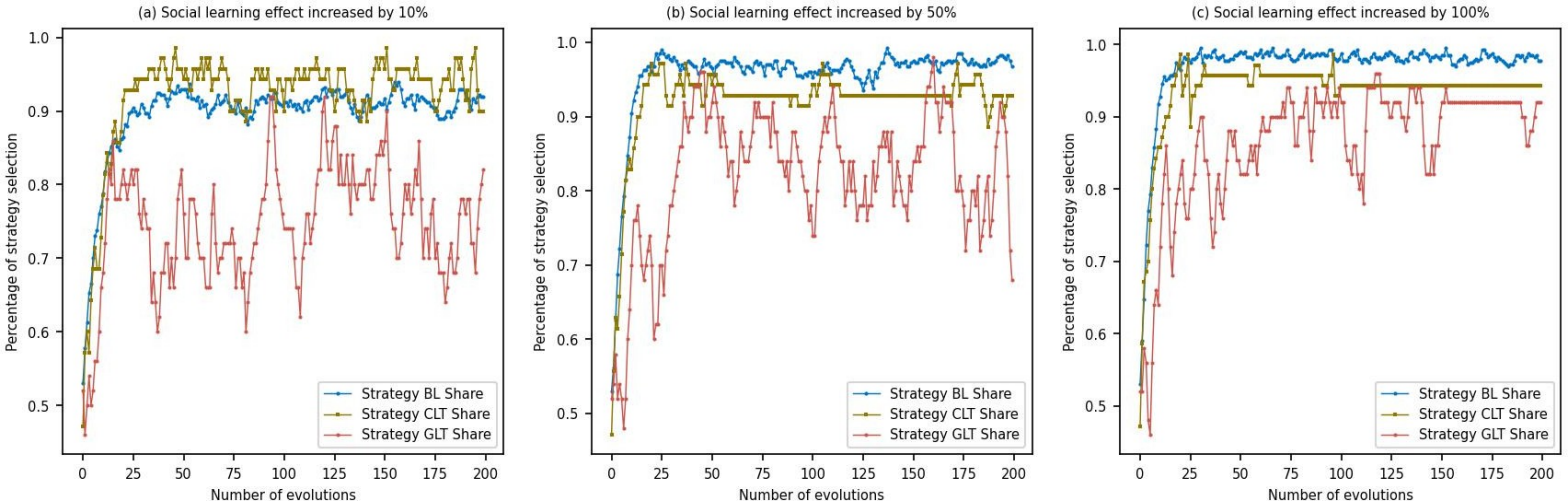
Social learning effect.

In this paper, the parameter *β*_*g*_ in Eq (9) is an exogenous parameter that serves to control the degree of social learning among agents. Specifically, a higher value of the parameter *β*_*g*_ implies that agents are more sensitive to changes in utility, making them more susceptible to the influence of others’ strategies and consequently adjusting their own choices. Therefore, in this study, we have increased the social learning effect by 10%, 50%, and 100% respectively, with the aim of observing the dynamics of equilibrium, as illustrated in Fig 9.

From the above figures, it can be observed that when the social learning effect increases by only 10% (Fig 9a), the proportion of consumers choosing the BL strategy is around 0.9, high-energy-consuming enterprises opting for the CLT strategy hovers around 0.95, and power companies choosing the GLT strategy fluctuate in the range of 0.7–0.8. As the social learning effect increases to 50% (Fig 9b), the proportion of consumers selecting the BL strategy reaches approximately 0.98, high-energy-consuming enterprises continue to favor the CLT strategy at around 0.95, and power companies fluctuate between 0.8–0.9 in favor of the GLT strategy. Notably, with an increase in simulation iterations, the variability in strategy proportions is significantly reduced compared to when the social learning effect was at 10%. When the social learning effect increases by 100% (Fig 9c), there is no significant change in the strategy choices of consumers, high-energy-consuming enterprises, and power companies. However, compared to scenarios with lower social learning effects, their strategy proportions reach equilibrium more rapidly. Fig 9a–9c reveal that as the social learning effect increases from 10% to 100%, although the gains for consumers, high-energy-consuming enterprises, and power companies show only marginal improvement, the primary change is the accelerated attainment of game equilibrium by all parties. When the social learning effect is low, consumers are more inclined towards traditional high-carbon products, and the willingness of high-energy-consuming enterprises and power companies to adopt low-carbon transformation strategies is limited, resulting in market instability and slower attainment of game equilibrium. However, as the social learning effect improves, consumers tend to choose low-carbon products, and businesses are more willing to embrace low-carbon transformation strategies. This leads to a quicker achievement of game equilibrium, and although the gains for all parties are modest, market stability and sustainability significantly increase. Therefore, social learning shortens the response time of each subject to the dynamic development of the market and activating market dynamics. This conclusion is in concordance with earlier research findings [56]. This has important economic implications for decision-makers and policymakers, encouraging the adoption of more sustainable strategies and advancing the goals of sustainable development.

## 5. Conclusion and policy implications

This paper explores the dynamics of multi-agent low-carbon transition decision-making within carbon market trading. By incorporating social learning into a network evolutionary game model, we offer a novel perspective on how various stakeholders adapt strategies to maximize utility during the low-carbon transition.

Key findings from simulation experiments include: (1) Carbon allowance allocation significantly impacts power generation enterprises’ willingness to engage in low-carbon transformation. (2) Higher prices for low-carbon products from high-energy-consuming enterprises boost enthusiasm for low-carbon transformation among all parties. (3) Green premium increases positively impact profits and utility but are subject to policy constraints and carbon prices. (4) Both direct and indirect carbon emissions negatively affect profits and utility for all entities. (5) Increasing social learning prompts a shift toward low-carbon strategies, accelerating game equilibrium and enhancing market stability and sustainability.

Implications for decision-making under carbon trading: (1) Governments should balance carbon quota allocation, ensuring power generation companies’ enthusiasm for low-carbon transformation is maintained. Attention should be given to high-energy-consuming enterprises and consumers, ensuring commitment to low-carbon practices. (2) Escalating prices of low-carbon products motivates all stakeholders to engage in low-carbon transformation, requiring governments and industries to capitalize on this trend. (3) Governments and industries should persistently optimize policies and market mechanisms to incentivize businesses for low-carbon transformations, meeting consumer demands for sustainable products. (4) Measures to encourage low-carbon transformation in high-energy-consuming enterprises, along with raising consumer awareness, are crucial for achieving carbon reduction and green development goals.

Our study differs from prior research that mainly examines the macro-level effects of carbon trading by focusing uniquely on the nuanced decision-making processes of market participants. We investigate how they respond to various policies and assess the impact of social learning within the market on decisions related to the transition to a low-carbon economy. This approach contributes to a deeper comprehension of carbon trading dynamics, uncovering micro-level intricacies often overlooked in earlier literature. However, it is important to acknowledge limitations, such as the necessity to consider regional and industry variations. This implies potential avenues for future research to explore the evolution mechanisms across diverse regions and industries, offering insights for governments to formulate targeted low-carbon policies.

## Supporting information

S1 Data (ZIP)
